# 

**Published:** 1904-11

**Authors:** 


SUBSCRIPTION $1.00

PUBLISHED MONTHLY

### ATLANTA JOURNAL-RECORD OF MEDICINE.

Successor to Atlanta Medical and Surgical Journal, Established 1855,
and Southern Medical Record, Established 1870.

INDEXED
S. G. O.

Vol. VI. Atlanta, Ga., November, 1904. No. 8.

